# Levels and determinants of ambulatory mobility in lower-limb prosthesis users from urban and rural Cambodia: a cross-sectional survey study

**DOI:** 10.1136/bmjopen-2025-101187

**Published:** 2025-07-25

**Authors:** Nerrolyn Ramstrand, Alan Maddock, Thearith Heang, Nil Ean, Sisary Kheng

**Affiliations:** 1 Department of Rehabilitation, Jönköping University, Jönköping, Sweden; 2 Department of Health Psychology, School of Population Health, Royal College of Surgeons in Ireland, Dublin, Ireland; 3 Exceed Worldwide, Phnom Penh, Cambodia; 4 Department of Prosthetics and Orthotics, National Institute of Social Affairs, Phnom Penh, Cambodia; 5 The Center for Trauma Care and Research Organization (CTRO), Phnom Penh, Cambodia; 6 Department of Psychology, Royal University of Phnom Penh, Phnom Penh, Cambodia

**Keywords:** Amputation, Surgical, REHABILITATION MEDICINE, Gait

## Abstract

**ABSTRACT:**

**Objectives:**

The majority of people globally who have undergone limb amputations are living in low- and middle-income countries. For those with lower-limb amputations, ambulatory mobility with a prosthesis is considered a key factor for achieving independent living; however, little is known of determinants of mobility for prosthesis users in low- to middle-income countries. In this study, we sought to assess levels of self-reported mobility in Cambodian prosthesis users and to identify determinants associated with their ambulant mobility.

**Design:**

Cross-sectional survey.

**Setting:**

Three secondary care centres located in urban and rural Cambodia.

**Participants:**

Adults receiving prosthetic services for a major lower-limb amputation.

**Primary and secondary outcome measurements:**

The Khmer version of the LCI-5 and the mobility dimension of the EuroQol five-dimensional five levels (EQ-5D-5L) were used as dependent variables, while personal, physical, psychological and social determinants served as independent variables. Associations were assessed using hierarchical and ordinal regression analyses.

**Results:**

347 participants completed the survey. Determinants that were negatively associated with mobility outcomes were as follows: being female, having an amputation due to dysvascular complications, using an above-knee prosthesis and reporting higher levels of psychological distress. Social determinants did not appear to have any major association with mobility outcomes.

**Conclusions:**

Findings highlight the multidimensional nature of mobility and suggest that future interventions may benefit from targeting female prosthesis users and those with diabetes or vascular disease. Mental health interventions addressing symptoms of anxiety and depression may also contribute to improved mobility outcomes. Social determinants explored in the study were not associated with significant changes in mobility scores. This may be due to high overall levels of mobility, a relatively homogenous group or failure to identify context-specific variables that impact on mobility outcomes.

**Strengths and limitations of this study**

- A broad selection of variables was investigated covering personal, physical, social and psychological factors that may affect mobility outcomes.
- Trained research assistants supported participants with literacy challenges in responding to the survey, reducing potential for response bias and making the study more inclusive.
- Mobility was assessed using self-reported measures and may not reflect the true physical capacity of participants.
- The cross-sectional design prevents the assessment of causal relationships between determinants and mobility outcomes.

## Introduction

 It is estimated that 65 million people globally are living with a limb amputation and that two-thirds of these people are residing in low- and middle-income countries.^1^ Underlying causes of amputation vary from region to region.^2^ While amputations resulting from peripheral vascular disease and/or diabetes (dysvascular amputations) are most common in high-income countries,^3 4^ trauma is generally the most common cause of amputation in low- and middle-income countries.^2 5^ Mobility is recognised as a key component in achieving and maintaining independent living following a lower-limb amputation. Higher levels of mobility are associated with improved quality of life and higher levels of general satisfaction following an amputation.^6 7^ Understanding the determinants of mobility in prosthesis users is key to improving outcomes for people with lower-limb amputations; however, little is known about mobility determinants for people living in low- to middle-income countries, such as Cambodia.

Mobility has been defined in various ways but is generally accepted to involve a change in body position from one location to another, whether it be on foot or through alternate means of transport.^8^ Over the past decades, a compelling body of evidence has been generated to suggest that mobility is a multidimensional construct, influenced by inter-related determinants.^9^,^11^ This is conceptualised in Webber *et al*’s^12^ comprehensive mobility framework, proposing five categories of determinants that can impact on mobility outcomes in older adults: financial, psychosocial, environmental, cognitive and personal life history.^12^ Inherent to this framework is the notion that mobility determinants are interrelated and that factors will have different levels of relevance depending on the context within which a person is living.

This paper focuses on ambulant mobility in people using lower-limb prosthetic devices. Achieving a high level of ambulant mobility with a prosthesis requires users to adapt to a range of scenarios, from relatively simple straight walking on level surfaces to more complex tasks, such as navigating uneven terrain, avoiding obstacles and ascending or descending stairs.^13^ Much of the existing literature evaluating determinants of ambulant mobility in prosthesis users has been conducted in high-income country contexts,^14^,^21^ with few studies investigating prosthesis users in low- to middle-income countries.^22^ To date, determinants identified as having a significant association with ambulant mobility include personal factors related to age, time since amputation, cause of amputation and prosthesis use,^1621^,^23^ as well physical factors related to pain^24^ and body weight.^18 20^ Other categories of determinants have received less attention, but psychological determinants such as self-esteem, anxiety and depression have been linked to levels of ambulant mobility in users of lower-limb prostheses,^19 20^ as well as the environment in which a person is living (urban/rural).^16^

Cambodia is a lower middle-income country with an estimated population of 17 million in 2024.^25^ The country has experienced some of the highest rates of amputation in the world,^26^ predominantly resulting from conflicts during the Cambodian civil war between 1967 and 1975 and the atrocities of the Khmer Rouge regime between 1975 and 1989.^27^ In the years following this period of violence, the prevalence of conflict-related injuries has declined, but amputations caused by road traffic accidents and medical conditions such as diabetes, infection and gangrene have increased.^28^ Although national records documenting amputation rates in Cambodia are not currently gathered, a review of data collected from three Cambodian prosthetic and orthotic clinics between 2005 and 2019 suggests that transtibial (TT) amputations represent the most common level of amputation, while the incidence of transfemoral (TF) amputations has been gradually rising.^28^ Prosthesis users in Cambodia have been reported to have a very high prevalence of psychological distress (anxiety and depressive symptoms) and post-traumatic stress disorder (PTSD) symptoms,^29^ likely linked to high levels of poverty, the country’s violent past and limited access to mental health support.^30^ Little is known of mobility levels of prosthesis users in Cambodia, although access to a prosthetic device is perceived to improve functional performance and levels of social inclusion.^30^

The aim of this study was to assess self-reported ambulant mobility in Cambodian prosthesis users and to identify determinants associated with ambulant mobility. The study addresses a critical gap in the literature by focusing on a lower middle-income country context and by simultaneously exploring a broad range of determinants.

## Methods

We conducted a cross-sectional survey with lower-limb prosthesis users in Cambodia. Data were collected from three prosthetic and orthotic clinics run by a non-governmental organisation (NGO), Exceed Worldwide, including one urban clinic (Phnom Penh) and two rural clinics (Kampong Som and Kampong Chhnang). Data collection took place between March 2023 and July 2024 as part of a larger study investigating the mental health of prosthesis and orthosis users in Cambodia.^31^ This study conforms to the principles outlined in the Declaration of Helsinki. Ethical approval was obtained from the National Ethics Committee for Health Research in Cambodia (#207 NEHCR). Potential participants were provided with oral information about the study. The first question of the survey required that participants check a box indicating that they understood the information provided and consented to participate.

### Participants

The study sample size was determined by inviting eligible clients presenting to all three prosthetic clinics the opportunity to participate over the 16 month period of testing. The final sample was subsequently dependent on the number of eligible clients available and willing to participate, rather than being predetermined by a formal sample size calculation. Participants were included if they were older than 18 years of age and could speak fluent Khmer, the national language of Cambodia. Those who attended clinics for prescription of a wheelchair or who attended the clinic but were not prescribed an assistive device were excluded. While the initial data collection involved users of either prosthetic and orthotic devices, data extracted for the present study included only participants who had undergone unilateral or bilateral lower-limb amputations and were fitted with one or more lower extremity prostheses, including TT, knee disarticulation (KD), TF or hip disarticulation prostheses (HD). Patients with partial foot prostheses were not included in the analysis. No restriction was placed on the cause of amputation.

### Data collection

Participants consenting to participate were requested to complete a survey in the Khmer language using a digital form generated in Qualtrics (Qualtrics, Provo, UT). Data collection took place onsite at the prosthetic and orthotic clinic, and clients with low levels of literacy were offered the opportunity to have the questions and response alternatives read to them by a research assistant, who also assisted in documenting participants’ responses in the Qualtrics questionnaire.

**Figure 1:**
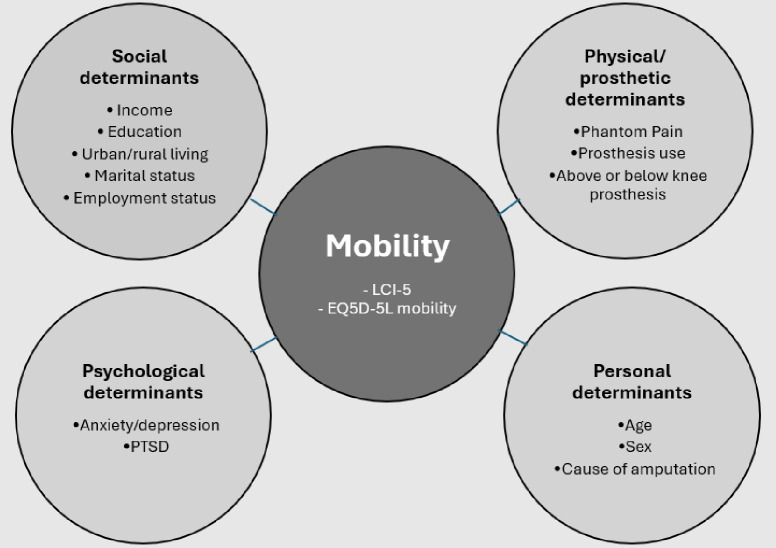
Schematic diagram of determinants explored in this study. EQ-5D-5L, EuroQol five-dimensional five levels; LCI-5, Locomotor Capabilities Index 5; PTSD, post-traumatic stress disorder.

Figure 1 presents an overview of mobility measures and determinants that were explored in this study. Due to time limitations with each participant, we chose to limit mobility measures to self-report instruments only. As there are currently only two self-report mobility measures translated and validated in the Khmer language, these were both used as dependent variables. They included (1) the Locomotor Capabilities Index (LCI-5)^32^ and (2) the mobility dimension of the EQ-5D-5L.^33 34^ The LCI-5 was selected as it focuses on the extent to which a person can perform specific movements and contains activities that have previously been identified as difficult to perform by Cambodian prosthesis users.^30^ The EQ-5D-5L was included as it focuses on perceived difficulties with general mobility and may have been more likely to capture effects of psychosocial factors on mobility outcomes.

Determinants of mobility included in the analysis represented a broad range of categories including personal, physical, social and psychological factors. Determinants were selected on the basis that they had been identified in previous research as being associated with mobility outcomes in prosthesis users.^14^,^2022 23^ Some previously identified factors were excluded as accurate self-report data on these factors were considered too difficult to collect. These included comorbidities such as kidney disease,^18^ prior re-vascularisation or vascular disease,^18 20^ hypertension and alcohol disorders.^19^ We also excluded prosthetic socket fit, need for replacement device,^20^ ability to don and doff a prosthesis,^20^ body weight^20^ and body mass index^18^ as these data were also considered too difficult to collect from a self-report survey in Cambodia. Previously unexplored variables that were considered important in the Cambodian context were added. These included PTSD symptoms, level of education, income and employment status.

### Mobility measures (dependent variables)

#### LCI-5

The LCI-5 is a self-report measure that was specifically designed to evaluate the ability of lower-limb prosthesis users to perform activities with their prosthetic device.^35^ The original English version comprises 14 activities rated on a five-point ordinal scale ranging from 0, unable to perform the activity, to 4, able to perform the activity without ambulation aids. The Khmer version of the LCI-5 has undergone extensive translation and psychometric testing^36 37^ and comprises only 10 items due to issues with covariance between some of the original items.^37^ Factor analysis performed on the Khmer version of the LCI-5 has demonstrated that items group into two subscales, each containing five items. One subscale represents activities that are considered basic to perform in the Cambodian context, while the other represents activities that are considered advanced. Higher scores reflect higher levels of mobility.

#### Mobility dimension of the EQ-5D-5L

The EQ-5D-5L is a self-report measure of health-related quality of life, covering five dimensions. The mobility dimension, comprising a single item, requires participants to rate their mobility from 1, I have no problems in walking around, to 5, I am unable to walk around. A systematic review evaluating the psychometric properties of the EQ-5D-5L has indicated that the instrument is reliable and valid and confirms that individual dimensions have moderate to strong correlations with other relevant measures.^34^ The ED-5D-5L has been translated into Khmer using a standardised translation protocol.^33^ Approval to use the instrument was obtained from the EuroQol research foundation (Rotterdam, Netherlands). In contrast to the LCI-5, lower scores on the EQ-5D-5L mobility dimension reflect higher levels of mobility.

#### Personal determinants

Personal determinants explored in this study included the age and sex (male, female) of prosthesis users as well as cause of amputation. Age was recorded using six ordered categories beginning from 18 to 24 years and ending with 64+ years. 64 was used as the starting point in the highest age category as this reflects retirement age in the Cambodian population. Categories for reporting the cause of amputation were trauma, infection, dysvascular complications, congenital, tumour and other.

#### Physical/prosthetic determinants

Response options for the type of prosthesis used by participants were dichotomised into two distinct categories: (1) below knee prostheses, including ankle disarticulation and TT prosthesis users, and (2) above-knee prostheses, including KD, TF and HD prostheses. People with bilateral lower-limb amputations were categorised by their most proximal level of amputation.

Phantom limb pain was evaluated with four ordered response alternatives (no pain, once per year, less than once per week or once per week or more). Device use was recorded using the prosthetic use score,^38^ where a score of 100 indicates that the device is worn every day for more than 15 hours.

#### Social determinants

Employment status was evaluated using the response alternatives—home-maker, business owner, employed and student. No distinction is made between part-time and full-time employment because, in the Cambodian context, work is typically informal, irregular and driven by necessity rather than structured job contracts. Income was evaluated by asking participants to indicate their average monthly income (US$) over the past 6 months, selecting from ordered categories. Our previous experience conducting research in the Cambodian context indicated that people are ashamed to reveal that they do not have any regular income and will instead respond to questions about income by stating that they ‘work in the home’ (understood by Cambodians to mean that they do not earn an income). For this reason, we included two categories, ‘no income’ and ‘work in the home’. These were subsequently collapsed into one category, ‘no income’. Marital status was assessed using four categories: single, married, divorced or widowed, while educational level of participants was assessed by requesting participants to indicate the highest level of education completed. Response alternatives were developed to align with the Cambodian education system (response alternatives—no education, primary school education (6 years), secondary school education (3 years), high school education (3 years) and college/university education (4 years). Finally, participants were requested to indicate their residential status by indicating if they lived in an urban setting (Phnom Penh or regional capital) or a rural setting.

#### Psychological determinants

Symptoms of anxiety and depression were evaluated using the Khmer version of the Kessler Psychological Distress Scale (K10), this 10 item scale reflects the likelihood that respondents are experiencing psychological distress.^39 40^ Total scores are created by adding the individual scores on each item and provide an indication of the individual’s emotional state. Scores under 20 indicate that the respondent is well, scores between 20 and 29 indicate mild to moderate distress and scores of 30 or over indicate the presence of a mental disorder.^39^ The K10 is available in Khmer,^41^ and previous work, involving prosthesis users in Cambodia, has shown that this version has a high level of internal consistency.^29^

PTSD symptoms were measured using the PC-PTSD-5.^42 43^ This instrument begins with a single question to establish if respondents have been exposed to traumatic events. Those who confirm exposure continue and are requested to complete five additional questions. Total scores over three are suggested as having optimal sensitivity and specificity in detecting PTSD symptoms.^44^ A Khmer version of the PC-PTSD-5 has been determined to have acceptable levels of internal consistency.^29^

### Data analysis

Survey responses were screened to remove records where the participant failed to respond to any of the questions and participant identifiers were removed and replaced with a code. If multiple responses were identified from the same client, suggesting they completed the questionnaire on multiple visits, data were only included the first time the questionnaire was completed. Categories where no responses were recorded were not included in regression analyses. These included the categories of ‘tumour’ and ‘infection’ as a cause of amputation (see table 1). Descriptive statistics were used to calculate frequencies and percentages of categorical and ordinal variables, while means and SD were calculated for continuous variables. A paired samples t-test was used to compare LCI-5 basic and advanced scores for the total sample. Separate hierarchical multiple regression analyses were conducted to examine associations between determinants and LCI-5 subscales (basic and advanced), while an ordinal regression analysis was conducted to examine associations between determinants and EQ-5D-5L mobility subscale scores. All analyses were performed using IBM SPSS Statistics 28 (IBM, Armonk, NY).

**Table 1:** Descriptive statistics

Variable	N(%)
Age	
18–24	19 (5.5)
25–34	28 (8.1)
35–44	60 (17.3)
45–54	67 (19.3)
55–63	114 (32.9)
64+	59 (17.0)
Sex	
Male	306 (88.4)
Female	40 (11.5)
Cause of amputation	
Trauma	309 (89.0)
Tumour	0 (0)
Infection	0 (0)
Congenital	21 (6.1)
Vascular	16 (4.6)
Other	1 (0.3)
Phantom pain	
Never	20 (5.8)
Once per year	82 (23.6)
Less than once per week	19 (5.5)
Once or more per week	4 (1.2)
Employment status	
Home-maker	18 (5.2)
Business owner	203 (58.5)
Employee	73 (21.0)
Unemployed	47 (13.5)
Student	4 (1.2)
Prosthesis type	
Below knee	242 (69.7)
Above knee	105 (30.3)
Average income	
No income	38 (11.0)
Work in the home	19 (5.5)
<150 US$	55 (15.9)
150–250 US$	91 (26.2)
250–400 US$	53 (15.3)
400+ US$	12 (3.5)
Do not wish to disclose	79 (22.8)
Marital status	
Single	37 (10.7)
Married	292 (84.1)
Divorced	9 (2.6)
Widowed	9 (2.6)
Education	
None	64 (8.4)
Primary	133 (38.3)
Secondary	83 (23.9)
High school	40 (11.6)
College/university	26 (7.5)
Residential status	
Urban	126 (37.2)
Rural	213 (62.8)
PC-PTSD-5	
No exposure to traumatic event	120 (34.6)
Exposure to traumatic event but score below threshold (3)	195 (56.2)
Exposure to traumatic event score above threshold (3)	32 (9.2)
K10 (psychological distress)	
Low	268 (77.2)
Moderate	74 (21.3)
High	5 (1.4)

PC-PTSD-5, five item primary care post-traumatic stress disorder.

Categories of determinants were entered into the hierarchical multiple regression analyses in four blocks. Personal determinants (age, sex and cause of amputation) were entered as block 1. Age was treated as a continuous variable,^45^ sex was dichotomised into two categories (male and female) with female entered as the reference variable. The cause of amputation was entered as polytomous variables with trauma used as the reference variable. Physical and prosthetic-related determinants (phantom pain, prosthesis use and type of prosthesis) were entered as block 2. Phantom pain and prosthetic use scores were treated as continuous variables, while the type of prosthesis was treated as a dichotomous variable with below-knee prostheses serving as the reference. Block 3 comprised social determinants and included average income, educational level, residential status, marital status and employment status. Income and education were treated as continuous variables. Residential status was dichotomised into urban or rural living, with urban living as the reference variable. Marital status was treated as a polytomous variable with ‘married’ as the reference variable, and employment status was treated as a polytomous variable with ‘employed’ used as the reference variable. The final block (block 4), containing psychological factors, comprised K10 and PC-PTSD-5 scores, which were both treated as continuous variables.

In the ordinal regression analysis, the score from the EQ-5D-5L mobility domain was entered as the dependent variable, while all predictors were entered in the same hierarchical manner as the previously described multiple regression analyses.

### Patient and public involvement

Patients and the public were not involved in the study design or implementation of this research.

## Results

### Descriptive

347 lower-limb prosthesis users completed the survey. Data related to the number of participants requested to participate were not completed at every site, and response rates were unable to be calculated. Table 1 summarises descriptive data for the sample. The majority of participants were male, TT prosthesis users (61%; n=213). 30% of males included in the sample wore TF prostheses (n=93), while 20% of females included in the sample wore TF prostheses. Trauma was the most common cause of amputation among participants (89%; n=309). 91% of males reported that they had undergone an amputation following trauma (n=281), while 67% of women reported having a traumatic amputation (n=27). The majority of respondents were aged between 55 and 63 (33%; n=114) with 17% (n=51) of male respondents being over retirement age compare to 20% (n=8) of women.

The average LCI-5 basic score was 19.64 (SD1.3; range 9–20) out of a total possible score of 20 and a Cronbach’s alpha of 0.76. The average LCI-5 advanced score was 18.37 (SD3.2; range 0–20) with a Cronbach’s alpha of 0.75. The difference between basic and advanced LCI-5 scores was statistically significant (t(344) = 8.93, p<0.001) with a moderate effect size (d=0.48 (95% CI: 0.37 to 0.59). Responses on the EQ-5D-5L indicated that 55.6% of participants (n=193) perceived no problems walking around, 38.6% (n=134) experienced a little trouble walking, 4.3% (n=15) experienced moderate walking problems and 0.9% (n=3) experienced severe walking problems. No participants reported that they were ‘unable to walk around’. The mean prosthetic use score was 84.14 (SD18.81). The mean K10 score was 15.59 (SD=4.97), and the mean PC-PTSD score was 1.43 (SD=1.61).

### LCI-5 results

A hierarchical multiple regression was run to determine if the addition of each category of determinants improved the prediction of LCI-5 basic subscores. Full details are presented in table 2. The full model containing all variables (model 4) was statistically significant, R^2^=0.184, F (19,202)=2.39, p=0.001, adjusted R^2^=0.107. The addition of physical/prosthetic determinants (model 2) and the addition of psychological determinants (model 4) led to a statistically significant increase in R^2^ (p<0.05). Addition of social determinants did not significantly contribute to a change in scores (p>0.05). A similar analysis was performed using LCI-5 advanced subscores (see table 3). Once again, the full model containing all variables (model 4) was statistically significant, R^2^=0.181, F (19,202)=2.351, p=0.001, adjusted R^2^=0.104, and the addition of physical/prosthetic determinants (model 2) as well as psychological determinants (model 4) led to a statistically significant increase in R^2^ (p<0.001).

**Table 2:** Hierarchical regression results for LCI-5 basic subscores

	LCI-5 basic							
	Model 1		Model 2		Model 3		Model 4	
	B	β	B	β	B	β	B	β
(Constant)	19.84**		19.07**		18.99**		19.65**	
Age	−0.02	−0.022	−0.055	−0.06	0.01	0.006	0.05	0.049
Sex (female)	−0.62*	−0.151	−0.616	−0.15*	−0.72*	−0.176	−0.79*	−0.194
Cause (ref: trauma)								
Dysvascular	−1.19**	−0.191	−1.093	−0.18**	−0.96*	−0.153	−0.79	−0.127
Congenital	0.33	0.059	0.256	0.047	0.27	0.050	0.32	0.059
Phantom pain			0.071	0.027	0.12	0.044	0.10	0.039
Prosthetic use			0.010	0.132*	0.01*	0.153	0.01*	0.170
Prosthesis (above-knee)			−0.373	−0.129	−0.33	−0.114	−0.28	−0.098
Income					−0.07	−0.099	−0.09	−0.134
Education					0.05	0.041	0.00	0.004
Residence (rural)					0.02	0.007	0.01	0.004
Marital status (ref: married)								
Single					0.06	0.014	0.16	0.037
Divorced					−0.42	−0.051	−0.44	−0.054
Widowed					0.14	0.017	0.21	0.026
Employment (ref: employee)								
Housewife					0.11	0.019	0.13	0.023
Business owner					−0.26	−0.097	−0.13	−0.047
Unemployed					−0.78*	−0.204	−0.71*	−0.185
Student					−0.34	−0.028	−0.52	−0.042
PTSD							0.20**	0.242
K10							−0.07**	−0.252
R2	0.06		0.097		0.13		0.184	
F	3.65**		3.302**		1.8*		2.39**	
∆R2	0.06		0.034		0.033		0.053	
∆F	3.65**		2.73*		0.77		6.58**	

*p<0.05; **p<0.001.

LCI-5, Locomotor Capabilities Index 5; PTSD, post-traumatic stress disorder.

**Table 3:** Hierarchical regression results for LCI-5 advanced subscores

	LCI-5 advanced							
	Model 1		Model 2		Model 3		Model 4	
	B	β	B	β	B	β	B	β
(Constant)	18.613**		20.629**		19.822**		20.864**	
Age	−0.005	−0.002	−0.121	−0.121	0.063	0.028	0.177	0.077
Sex (female)	−1.949**	−0.193	−2.163	−2.163**	−2.504**	−0.248	−2.709**	−0.268
Cause (ref: trauma)								
Dysvascular	−1.983	−0.129	−2.395	−2.395*	−2.229*	−0.145	−1.887	−0.123
Congenital	1.522	0.113	1.292	1.292	0.997	0.074	1.133	0.084
Phantom pain			0.635	0.635	0.600	0.091	0.527	0.080
Prosthetic use			−0.030	−0.030*	−0.028*	−0.155	−0.025	−0.136
Prosthesis (above-knee)			−1.289	−1.289**	−1.214*	−0.171	−1.133*	−0.160
Income					0.035	0.021	−0.032	−0.019
Education					0.078	0.028	−0.016	−0.006
Residence (rural)					0.081	0.012	0.113	0.017
Marital status (ref: married)								
Single					1.078	0.103	1.292	0.124
Divorced					−0.499	−0.025	−0.627	−0.031
Widowed					0.630	0.031	0.730	0.036
Employment (ref: employee								
Housewife					0.612	0.042	0.623	0.043
Business owner					−0.713	−0.109	−0.377	−0.057
Unemployed					−0.798	−0.085	−0.702	−0.075
Student					−0.666	−0.022	−0.962	−0.032
PTSD score							0.501**	0.249
K10							−0.135**	−0.197
R2	0.058		0.116		0.139		0.181	
F	3.357*		4.024**		1.935*		2.351**	
∆R2	0.058		0.058		0.023		0.042	
∆F	3.357*		3.087**		3.121		3.059**	

*p<0.05; **p<0.001.

LCI-5, Locomotor Capabilities Index 5; PTSD, post-traumatic stress disorder.

### EQ-5D-5L results

Results from the ordinal regression with EQ-5D-5L mobility dimension data are presented in table 4. The models in this analysis did not provide a significantly better fit to the data when compared with the intercept-only model (p>0.05), suggesting that the combined set of determinants in each model did not provide strong explanatory power for the mobility outcome. Despite this, there was a significant negative association between being female and EQ-5D-5L mobility domain scores (b=−1.438, SE=0.530, p=0.007, 95% CI: −2.477 to −0.399), suggesting that females were less likely to report problems walking around. A significant positive association was also observed between K10 scores and EQ-5D-5L mobility data (b=0.103, SE=0.034, p=0.003, b = 0.103, SE=0.034, p=0.003, b=0.103, SE=0.034, p=0.003, 95% CI: 0.035 to 0.170), indicating that higher K10 scores were associated with more self-reported problems walking around.

**Table 4:** Ordinal regression results EQ-5D-5L mobility domain scores

	EQ-5D-5L Mobility domain							
	Model 1		Model 2		Model 3		Model 4	
	β	SE	β	SE	β	SE	β	SE
(Threshold)								
Mobility=1	−0.032	1.413	0.984	1.844	18.452**	2.503	21.414**	2.853
Mobility=2	2.548	1.427	3.571	1.860	21.104**	2.525	24.143**	2.883
Mobility=3	4.403**	1.564	5.427	1.968**	22.969**	2.607	26.022**	2.958
Age	−0.067	0.102	−0.007	0.116	0.021	0.134	0.024	0.139
Sex (female)	−1.197**	0.467	−1.181*	0.467*	−1.460**	0.519	−1.438**	0.530
Cause (ref: trauma)								
Dysvascular	−0.415	0.753	−0.369	0.761	−0.612	0.804	−0.398	0.839
Congenital	1.418	1.180	1.167	1.215	0.906	1.271	1.101	1.389
Phantom_pain			0.292	0.330	0.433	0.342	0.458	0.347
Prosthetic use			0.005	0.008	0.009	0.008	0.012	0.009
Prosthesis (above-knee)			−0.186	0.324	−0.102	0.338	−0.029	0.345
Income					−0.061	0.110	−0.072	0.112
Education					0.124	0.154	0.176	0.158
Residence (rural)					−0.250	0.320	−0.396	0.334
Marital status (ref: married)								
Single					−0.463	0.576	−0.315	0.583
Divorced					1.362	0.940	1.704	1.032
Widowed					0.765	0.846	1.047	0.872
Employment (ref: employee)								
Housewife					−0.011	0.836	−0.005	0.858
Business owner					0.071	0.397	0.205	0.410
Unemployed					−0.519	0.614	−0.188	0.631
Student					16.185	0.000	15.810	0.000
PTSD_score							−0.134	0.114
K10							0.103*	0.034
−2 Log likelihood intercept	374.595		374.595		374.595		374.595	
−2 Log likelihood final	365.473		364.052		355.968		346.901	
X^2^	9.121		10.542		18.627		27.694	

*p<0.05; **p<0.001.

Mobility = 1 reflects the boundary between ‘no problems walking around’ and ‘little trouble walking around’. Mobility = 2 reflects the boundary between ‘little trouble’ and ‘moderate trouble walking around’. Mobility = 3 reflects the boundary between ‘moderate’ and ‘severe problems walking around’.

EQ-5D-5L, EuroQol five-dimensional five levels; PTSD, post-traumatic stress disorder.

## Discussion

Mobility is a multidimensional construct influenced by a broad range of determinants. The extent to which specific determinants impact on mobility outcomes is likely influenced by the broader country context. This study addressed a gap in scholarly literature by investigating determinants of ambulatory mobility in a low- to middle-income country, Cambodia, and by simultaneously investigating determinants spanning personal, physical, social and psychological dimensions. Results from the Khmer version of the LCI-5 indicated that being female and having an amputation due to dysvascular complications were associated with poorer mobility outcomes and that being an above-knee prosthesis user was associated with poorer mobility outcomes than being a below-knee prosthesis user. These results were not observed when the EQ-5D-5L mobility dimension was investigated as a mobility measure. Psychological determinants included in the study were identified as having significant associations with both ambulatory mobility outcomes, while social determinants did not appear to play a major role. Results from this study should not be interpreted as causal due to the cross-sectional design of the study.

The sample of participants recruited for this study is considered to be representative of the general population of people accessing prosthetic services in Cambodia. Our sample, comprising 88% male participants, is consistent with proportions reported from longitudinal data involving over 7000 clients attending the same three clinics as those investigated in the present study.^28^ Also consistent with our sample are reports of traumatic injury being the most common cause of amputation and the majority of prosthesis users in Cambodia being under 60 years of age.^28^ The disproportionate number of female prosthesis users reported in this and previous work, as well as the relatively low numbers of prosthesis users aged over 65 years, is of interest. While this is likely due to the large number of traumatic amputation, including landmine accidents, which are known to affect men more than women, it is unclear if these figures may also reflect an inequity in access to prosthetic services.^30 46^ At present, there is no accurate data from Cambodia reporting overall amputation prevalence relative to the proportion of individuals who ultimately receive prosthetic devices. Collecting and analysing amputation prevalence data from Cambodian hospitals will be necessary in the future to evaluate equitable service delivery. Such national statistics would also allow researchers to determine the extent to which this study’s findings can be transferred to the general population.

Levels of ambulatory mobility reported by participants in this study were surprisingly high in comparison to reports from high-income countries.^47^ While differences in LCI-5 factor loadings do not allow direct comparisons across countries,^37^ the large majority of Cambodian prosthesis users reported few difficulties in moving around. High mobility levels in this study sample may again reflect inequality in access to prosthetic services in Cambodia, with those who have comorbid medical conditions and poorer general health unable to access prosthetic clinics. High levels of mobility may also be attributable to the large number of participants with traumatic amputations, a group known to have higher levels of mobility than those whose amputations result from diabetes or peripheral vascular disease.^23 48^ This aligns with our findings, where participants with trauma-related amputations were more likely to have favourable mobility outcomes on the LCI-5 when contrasted to those whose amputations were associated with diabetes or vascular disease.

Among the sample of individuals investigated in the present study, being female was associated with poorer mobility outcomes than being male. This is consistent with findings from high-income-country contexts, employing both objective^49 50^ and self-report measures.^51^ In previous work, the disparity between females and males has been linked to the fact that women undergo more TF amputations than men.^52^ This does not appear to be the case in the present sample as women had a lower proportion of TF amputations than men. In the current study, the proportion of men and women over retirement age was relatively similar, so age is not unlikely to offer an explanation for the observed difference in mobility outcomes.

Previous studies have highlighted age as a significant factor influencing prosthesis users’ mobility, reporting a significant decrease in mobility with advancing age.^16 18 22^ Interestingly, our study did not identify such a relationship. This is likely due to the relatively young demographic who are accessing prosthetic services in Cambodia. Higher levels of mobility were reported by participants using below-knee prostheses, a result consistent with previous findings.^53 54^ This is unsurprising given the mechanical advantage offered by longer residual limbs.

Results related to the psychological determinants of anxiety and depression, estimated using the K10, revealed a significant negative association in both LCI-5 domains and a significant positive association for the EQ-5D-5L. Reflecting on the fact that lower LCI-5 scores represent less capability to perform mobility tasks while higher scores on the EQ-5D-5L represent more perceived mobility problems, combined results suggest that higher levels of psychological distress may be associated with lower levels of mobility. While the effect sizes for the K10 were modest on a per-unit basis, the result becomes more impactful when considering larger changes in K10. This, coupled with the finding that over 20% of participants reported moderate or high psychological distress, highlights a need for mental health support of prosthesis users in Cambodia. While individuals reporting high levels of psychological distress should be referred directly to a clinical psychologist, there is evidence that peer support programmes may serve as a useful intervention in those reporting moderate psychological distress. A recent study provided evidence of large reductions in the K10 scores of Cambodian prosthesis users who participated in a mental health peer support programme.^55^

When exploring the relationship between PTSD and mobility, a significant positive association was observed with results from the LCI-5 domains, while no significant association was recorded between the EQ-5D-5L and scores on the PC-PTSD-5. The positive association identified between PC-PTSD-5 scores and LCI-5 domains is challenging to interpret. This result seems to suggest that higher levels of PTSD are associated with higher levels of mobility. It is possible, however, that the low numbers of participants with probable PTSD in this study (n=32) may have led to a spurious finding in this instance.

While we aimed to cover a broad range of determinants in this study, it is important to acknowledge that the proportion of variance explained by the regression models was low. This may be due to the overall high levels of mobility reported by participants, which would likely reduce the explanatory power of the determinants in the regression models. Similarly, the relatively homogeneous sample of participants may have resulted in limited variation in the determinants that were investigated, reducing their apparent impact on mobility. It should also be acknowledged that some important variables may have been omitted. These could include physical factors related to balance, strength and range of motion, as well as social factors, specific to the Cambodian context. Examples include ease of access to health services, quality of services provided and experiences of social exclusion.

Two different measures of mobility were used in this study, LCI-5, a tool specifically designed for prosthesis users, and the mobility domain of the EQ-5D-5L, a generic outcome measure. While significant associations were observed for individual determinants with both outcome measures, overall models only improved predictions for LCI-5 scores. This finding is likely due to the specificity of the LCI-5 in capturing mobility challenges specific to prosthesis users. While other measures of mobility, specifically designed for prosthesis users, have been developed and may prove to be more appropriate for use on people with high levels of function,^56^ the use of any additional measure would require substantial work to translate and culturally validate the tool in the Cambodian context.

### Limitations and recommendations

One limitation of this study is the skewed distribution of some determinants (eg, marital status; K10 scores). In cases where the determinant was entered into the model as a continuous variable (eg, K10 scores), the uneven distribution may have limited our ability to detect robust associations with mobility outcomes. Similarly, categorical variables with uneven group sizes may have led to unstable parameter estimates for underrepresented categories, increasing the risk of wide CIs and reduced statistical power. While efforts were made to check model assumptions and interpret findings with caution, future research with a larger sample is recommended to clarify the role of these determinants in the observed associations.

We also acknowledge that interrelationships between determinants investigated in this study may have affected the stability of regression coefficients, limiting our ability to interpret the effects of individual determinants with confidence. We recommend that future studies include larger and more stratified samples to explore these complex relationships in greater depth.

The use of sophisticated prosthetic components, such as microprocessor-controlled knees, has been demonstrated to affect mobility outcomes in lower-limb prosthesis users.^57^,^59^ This relationship was not investigated in this study. While components may have influenced mobility outcomes in our sample, we argue that there is limited variation in the types of components prescribed in Cambodia and that the effects would have been minimal. Prescription of sophisticated components is rare in Cambodia due to prohibitively high costs.

The LCI-5 was selected for this study as it incorporated items that have previously been identified by Cambodian prosthesis users as challenging to perform.^30^ Both the LCI-5 and the mobility domain of the EQ-5D have been translated into Khmer and validated for use in the Cambodian context. While these self-report measures provide an accurate reflection of prosthesis users’ perceptions of their mobility and allow researchers to identify specific factors that are perceived as more challenging to perform, results may not reflect performance on objective measures.^60^ Given the high levels of mobility in the sample included in this study, quantifiable objective measures may have been more effective in detecting subtle differences in mobility associated with the determinants under investigation. In future studies, it would be useful to combine both self-report and performance measures of mobility in the Cambodian context.

Cambodia’s cultural and social situation as well as environmental influences (geographical terrain and climate) may uniquely shape the experiences of prosthesis users in the country and subsequently limit the generalisability of findings. It is important that future research explores mobility determinants in diverse settings to validate and expand on findings from this work.

Our study employed a cross-sectional design which does not allow us to establish causal relationships between the determinants examined and ambulatory mobility, nor does the design allow us to account for temporal changes in mobility, health status or other contextual factors. Future research, employing longitudinal study designs, is subsequently recommended.

## Conclusion

By examining personal, physical, psychological and social factors, this study identified determinants that affect ambulatory mobility outcomes of prosthesis users in a unique context, Cambodia. Key findings suggest that being female, having an amputation due to diabetes or vascular disease and using an above-knee prosthesis may be associated with poorer mobility outcomes. Additionally, psychological factors such as anxiety and depression significantly influence mobility outcomes. Social determinants appear to play a less prominent role. Future research should explore potential disparities in access to prosthetic services and should aim to validate findings in other low- and middle-income settings. Addressing these gaps will help ensure equitable and effective support for prosthesis users worldwide.

## Data Availability

Data from this study are available upon reasonable request.
