# Supplementary material for: Levels and determinants of ambulatory mobility in lower-limb prosthesis users from urban and rural Cambodia: a cross-sectional survey study
Source: BMJ Open. 2025 Jul 25;15(7):e101187. doi: 10.1136/bmjopen-2025-101187 (PMC12306211; doi:10.1136/bmjopen-2025-101187)
Supplement: Supplementary file 1 [file bmjopen-2025-101187.reviewer_comments.pdf]

PEER REVIEW HISTORY

BMJ Open publishes all reviews undertaken for accepted manuscripts. Reviewers are asked to complete a checklist review form and are provided with free text boxes to elaborate on their assessment. These free text comments are reproduced below.

ARTICLE DETAILS

Title (Provisional)

Levels and determinants of ambulatory mobility in lower limb prosthesis users from urban and rural Cambodia – A cross-sectional survey study.

Authors

Ramstrand, Nerrolyn; Maddock, Alan; Heang, Thearith; Ean, Nil; Kheng, Sisary

---

VERSION 1 - REVIEW

**Reviewer**	**1**
**Name**	**Worsley, Peter**
**Affiliation**	**University of Southampton, School of Health Sciences**
**Date**	**02-Apr-2025**
**COI**	**None**

This is an interesting manuscript exploring an important aspect of outcomes in individuals with amputation. There are clear strengths in the cross section of participants who were reached in Cambodia. I have a few comments for the authors to address.

1. How representative is the sample of individuals who have access to the NGO clinics? Does this create some selection bias when considering the transferability to the general population of Cambodia.

2. Are the demographics of the respondents reflective of the three clinics active prosthetic users and those who access the NGO clinics.

3. There is a large skew in many of the outcomes measures e.g. K10, what are the implications when conducting the regression statistics?

3. Proportion of variance explained in the model is very low. What do you think you missed?

4. There appear to be major confounders, for example those with amputation causes by peripheral vascular disease are more likely to have a number of comorbidities and history of immobility compared to those with a history of trauma. This may also link to higher levels of psychological distress. Indeed, there is high potential for unstable regression weights and difficulties in interpreting complex relationships.

5. You've highlighted that the cross sectional nature of the study limits any causal analysis and temporal trends. This should be better reflected in the tone of the discussion, and interpretation of the regression analysis.

6. More figures would be beneficial, to supplement the tables provided.

**Reviewer**	**2**
**Name**	**Clemens, Sheila M.**
**Affiliation**	**Florida International University**
**Date**	**16-Apr-2025**
**COI**	**None**

Thank you for the opportunity to review this interesting manuscript. The authors are reporting on an important area in prosthetic rehabilitation, and one that is rarely examined; recovery in lower-income countries. The article is well-written and interesting. However, I feel the authors could do better to expand on their reasoning as to why social determinants did not affect outcomes, as we often see in more developed nations. Additionally, some definitions are missing or should be clarified as they may impact outcomes and aid in clarification for the reader.

Abstract

• The Conclusions section should be reworked once other comments are addressed to reflect changes to the manuscript regarding a clearer rationale of social determinants, etc.

Introduction

• P6, L10: Can the authors provide a prevalence estimate, possibly by cause, of amputations in Cambodia specifically? It would strengthen the rationale for the study.

Methods

• P7, L18: The authors should provide reasoning why only self-reported measures of mobility were used and no performance-based measures of mobility were included in the study. This would be especially pertinent since data was collected in-person with the study participants.

• P7, L30: “board” is a typo, it should be “broad”

• P9, L13: The income categories are somewhat confusing. Did “employed” include any type of paid work, whether it was 1 hour or 40 hours per week? The level of employment (e.g., full time, part time, etc.) have been shown to contribute differently to recovery after amputation (those working full time exhibit significantly better mobility outcomes). If the authors are able to look more closely at the breakdown of employment status, it may elucidate some results that are currently being undetected. Additionally, was “homemaker”

an income category? As written, it appears it may have been. Please consider reworking this area for clarity.

• P9, L25: Were there definitions of “urban” and “rural” provided to the participants? Urban and rural environments need to be more clearly defined by the authors in this manuscript.

Results

• P10, L58: Was the difference in LCI-5 basic and advanced scores examined by amputation level, sex, etc.? This seems unclear leading to confusion as to what effect the effect size is reporting. Please add additional information for clarification.

• Table 1: “Housewife” is listed as an employment status. This should be edited to reflect “homemaker” as written in the text (additionally, this indicates that only females would be classified in this category). Additionally, listing “housework” as an income category seems inappropriate, as this is a type of work. And since the authors stated that this category was subsequently collapsed into “no income,” I think it is inappropriate to list it as an income category.

• Table 3 has the wrong title. It should read for “advanced” subscores.

• I assume since the EQ5D-5L has 5 mobility categories, yet only 3 are presented in Table 4, that 2 of the mobility categories weren’t chosen by study participants? If this is so, please mention this in the Results as it leaves the reader looking for the other 2 categories. Additionally, the word “knee” is missing from above-knee.

Discussion

• There is sufficient previous research establishing that women with lower limb amputations score lower on mobility outcomes compared to men (some examples: Gaunaurd I, Kristal A, Horn A, Krueger C, Muro O, Rosenberg A, Gruben K, Kirk-Sanchez N, Pasquina P, Gailey R. The utility of the 2-minute walk test as a measure of mobility in people with lower limb amputation. Archives of physical medicine and rehabilitation. 2020 Jul 1;101(7):1183-9; Cimino SR, Vijayakumar A, MacKay C, Mayo AL, Hitzig SL, Guilcher SJ. Sex and gender differences in quality of life and related domains for individuals with adult acquired lower-limb amputation: a scoping review. Disability and rehabilitation. 2022 Oct 23;44(22):6899-925; Clemens SM, Kershaw KN, Bursac Z, Lee SP. Association of race, ethnicity, and gender to disparities in functional recovery and social health after major lower limb amputation: a cross-sectional pilot study. Archives of physical medicine and rehabilitation. 2024 Feb 1;105(2):208-16.). It would potentially behoove the authors to examine some of the previous work and apply it to their findings in efforts to explain how their results regarding gender differences align with previous work.

• There is the possibility that the LCI was inappropriate for assessing mobility in this high functioning patient population (average scores appeared high). The recording of performance-based outcome measures and/or the use of a potentially more appropriate self-reported measure of mobility (i.e., PLUS-M) may have captured different results. The

authors should consider adding rationale regarding the appropriateness of outcome choice and potentially how the inclusion of performance-based measures may have resulted in different results.

• A better rationale of why social determinants did not impact mobility is warranted. Were there clear differences in urban and rural classifications? Again, definitions of these two environments within Cambodia would be beneficial. And possible differences in employment status (full time vs. part time, etc.) could have impacted the results, as observed in previous studies (Clemens SM, Kershaw KN, McDonald CL, Darter BJ, Bursac Z, Garcia SJ, Rossi MD, Lee SP. Disparities in functional recovery after dysvascular lower limb amputation are associated with employment status and self-efficacy. Disability and Rehabilitation. 2023 Jul 3;45(14):2280-7). These gaps in rationale should be expanded upon in a revised manuscript.

VERSION 1 - AUTHOR RESPONSE

Reviewer 1

1. How representative is the sample of individuals who have access to the NGO clinics? Does this create some selection bias when considering the transferability to the general population of Cambodia.

- We have addressed this issue at the end of the second paragraph of the discussion where we indicate that there is currently no accurate data from Cambodia reporting overall amputation prevelance relative to the proportion of individuals who ultimately receive prosthetic devices. To further clarify this point we have added test in the background section indicating that records on the total number and types of amputations performed are not available, as well as a comment in the discussion related to transferability to the general population.

2. Are the demographics of the respondents reflective of the three clinics active prosthetic users and those who access the NGO clinics.

- This issue has been addressed in the second paragraph of the discussion were we compare demographics of respondents in the present study to those reported in a previously published paper (n=7000). We have edited the text to make is clear that both studies collected data from the same three clinics.

3. There is a large skew in many of the outcomes measures e.g. K10, what are the implications when conducting the regression statistics?

- This is a fair comment. To address this issue we have been transparent in reporting the response rates of all sub-categories in table 1. We have also added a paragraph to our limitations and recommendations section to highlight the potential implications of having skewed determinants.

3. Proportion of variance explained in the model is very low. What do you think you missed?

- We have now made this fact clearer in the limitations section where we discuss variables we that were omitted but may be of potential relevance.

4. There appear to be major confounders, for example those with amputation causes by peripheral

vascular disease are more likely to have a number of comorbidities and history of immobility compared to those with a history of trauma. This may also link to higher levels of psychological distress. Indeed, there is high potential for unstable regression weights and difficulties in interpreting complex relationships.

- We have added a paragraph to our limitations and recommendations section to address this issue.

5. You've highlighted that the cross sectional nature of the study limits any causal analysis and temporal trends. This should be better reflected in the tone of the discussion, and interpretation of the regression analysis.

- We have edited the discussion to reflect the reviewers comment.

6. More figures would be beneficial, to supplement the tables provided.

- We chose to include tables rather than figures in this manuscript as we felt that it made the result more transparent and allowed readers to fully interpret the model outcomes. Given the low model fit and non-significant associations between determinants and mobility outcomes, graphical representations (e.g. scatter plots or coefficiant plots) would not reveal additional patterns beyond what is already apparent in the tables. In addition, including figures might inadvertantly suggest stronger trends than are warrented.

Reviewer: 2

Thank you for the opportunity to review this interesting manuscript. The authors are reporting on an important area in prosthetic rehabilitation, and one that is rarely examined; recovery in lower-income countries. The article is well-written and interesting. However, I feel the authors could do better to expand on their reasoning as to why social determinants did not affect outcomes, as we often see in more developed nations. Additionally, some definitions are missing or should be clarified as they may impact outcomes and aid in clarification for the reader.

Abstract
• The Conclusions section should be reworked once other comments are addressed to reflect changes to the manuscript regarding a clearer rationale of social determinants, etc.
Introduction

- We have reworked the conclusion to reflect changes to the manuscript.

• P6, L10: Can the authors provide a prevalence estimate, possibly by cause, of amputations in Cambodia specifically? It would strengthen the rationale for the study.

- As mentioned in our discussion, there are currently no overall amputation prevelance rates available in Cambodia. We have added some text to the introduction section to reflect this fact.

Methods
• P7, L18: The authors should provide reasoning why only self-reported measures of mobility were used and no performance-based measures of mobility were included in the study. This would be especially pertinent since data was collected in-person with the study participants.

- As limited time was available with each patient we chose to limit mobility measures to self-report instruments only. This is now reflected in the methods section.

• P7, L30: “board” is a typo, it should be “broad”

- This typo has been corrected.

• P9, L13: The income categories are somewhat confusing. Did “employed” include any type of paid work, whether it was 1 hour or 40 hours per week? The level of employment (e.g., full time, part time, etc.) have been shown to contribute differently to recovery after amputation (those working full time exhibit significantly better mobility outcomes). If the authors are able to look more closely at the breakdown of employment status, it may elucidate some results that are currently being undetected. Additionally, was “homemaker” an income category? As written, it appears it may have been. Please consider reworking this area for clarity.

- While we understand the reviewers comment it is important to consider the Cambodian context when reflecting on this issue. Poor people in Cambodia do not distinguish between part-time and full-time employment because their work is typically informal, irregular, and driven by necessity rather than structured job contracts. Many are engaged in farming, casual labor, street vending, or other types of informal work where hours are flexible, inconsistent, or tied to seasonal demands. As such, the concept of “part-time” versus “full-time” work is not familiar to them. We have added a sentence to the methods section ”social determinants” to refect this issue.
- Within Cambodian culture it is considered shameful if an individual does not have a job. For this reason people do not admit that they are unemployed rather they will indicate that they are a homemaker. This issues has been further clarified in the social determinants sub-section.

• P9, L25: Were there definitions of “urban” and “rural” provided to the participants? Urban and rural environments need to be more clearly defined by the authors in this manuscript.

- This has been further clarified in the methods section. Urban refered to Phnom Penh or regional capitals while all other regions were classified as rural.

Results

• P10, L58: Was the difference in LCI-5 basic and advanced scores examined by amputation level, sex, etc.? This seems unclear leading to confusion as to what effect the effect size is reporting. Please add additional information for clarification.

- In the descriptive results, LCI-5 basic and advanced scores were compared for the total sample. We did not explore differences for each determinant as this was explored in the regression models. We have clarified this in the data analysis section of the manuscript.

• Table 1: “Housewife” is listed as an employment status. This should be edited to reflect “homemaker” as written in the text (additionally, this indicates that only females would be classified in this category). Additionally, listing “housework” as an income category seems inappropriate, as this is a type of work. And since the authors stated that this category was subsequently collapsed into “no income,” I think it is inappropriate to list it as an income category.

- We have edited housewife to homemaker.
- While we understand the reviewers comment that it is a little odd to list housework as an income category we argue that within the Cambodian context this is necessary as, due to the shame associated with not having any income, people will instead say that they work in the home. This has been made clearer in the social determinants sub-section.

• Table 3 has the wrong title. It should read for “advanced” subscores.

- Thanks for picking up this error, we have corrected the title.

• I assume since the EQ5D-5L has 5 mobility categories, yet only 3 are presented in Table 4, that 2 of the mobility categories weren't chosen by study participants? If this is so, please mention this in the Results as it leaves the reader looking for the other 2 categories.

- The mobility dimension of the EQ5D-5L comprises of only one item with 5 rating options. We reported results for 4 of the options and have now added a sentence indicating that the 5th option 'unable to walk around' was not selected by any participants.
- In table 4 only three categories appear because these are the thresholds between the categories, not the categories themselves. In logistic regression the model estimates three cut-points (thresholds) along the mobility scale where the probability shifts from one category to the next: **Threshold 1**: boundary between Category 1 and 2 (No problems ↔ Little trouble); **Threshold 2**: boundary between Category 2 and 3 (Little trouble ↔ Moderate);**Threshold 3**: boundary between Category 3 and 4 (Moderate ↔ Severe). We have added text in the title of the table to make this clearer.

Additionally, the word "knee" is missing from above-knee.

- This has been corrected in the table.

Discussion

• There is sufficient previous research establishing that women with lower limb amputations score lower on mobility outcomes compared to men (some examples: Gaunaurd I, Kristal A, Horn A, Krueger C, Muro O, Rosenberg A, Gruben K, Kirk-Sanchez N, Pasquina P, Gailey R. The utility of the 2-minute walk test as a measure of mobility in people with lower limb amputation. Archives of physical medicine and rehabilitation. 2020 Jul 1;101(7):1183-9; Cimino SR, Vijayakumar A, MacKay C, Mayo AL, Hitzig SL, Guilcher SJ. Sex and gender differences in quality of life and related domains for individuals with adult acquired lower-limb amputation: a scoping review. Disability and rehabilitation. 2022 Oct 23;44(22):6899-925; Clemens SM, Kershaw KN, Bursac Z, Lee SP. Association of race, ethnicity, and gender to disparities in functional recovery and social health after major lower limb amputation: a cross-sectional pilot study. Archives of physical medicine and rehabilitation. 2024 Feb 1;105(2):208-16.). It would potentially behoove the authors to examine some of the previous work and apply it to their findings in efforts to explain how their results regarding gender differences align with previous work.

- We have added a paragraph comparing our observed gender differences to previous work but wish to highlight that underlying explainations for the difference are likely differenct in the Cambodian context. While previous work in high income country contexts has linked gender differences to more women with TF amputations this is not the case in our results, where men were found to have a greater proportion of TF amputation when compared to women. In oder to discuss this point it was necessary for us to also add additional text to the results section.

• There is the possibility that the LCI was inappropriate for assessing mobility in this high functioning patient population (average scores appeared high). The recording of performance-based outcome measures and/or the use of a potentially more appropriate self-reported measure of mobility (i.e., PLUS-M) may have captured different results. The authors should consider adding rationale regarding the appropriateness of outcome choice and potentially how the inclusion of performance-based measures may have resulted in different results.

- Once again cultural context is very important when addressing this issue. In order to use other measures they need to be validated in the local context and language. To date LCI-5 and EQ5D-5L are the only self-report mobility measures validated in the Khmer language and in the Cambodian context. We have added text to make this clearer in the methods and discussion. While PLUS-M is a great tool in the high income country context it is not

translated or validated into Khmer. It should be recognised that the English version of Plus-M includes concepts that are completly foreign to people living in rural Cambodia. For example many prosthsis users from rural areas have never seen a curb, they produce their own food and do not shop in supermarkets and they do not have driveways. As such much work would be required to culturally adapt PLUS.M before it is used in the Cambodian context.

- To address the reviewers comment on performance based measures we have added text to the limitations section.

• A better rationale of why social determinants did not impact mobility is warranted. Were there clear differences in urban and rural classifications? Again, definitions of these two environments within Cambodia would be beneficial. And possible differences in employment status (full time vs. part time, etc.) could have impacted the results, as observed in previous studies (Clemens SM, Kershaw KN, McDonald CL, Darter BJ, Bursac Z, Garcia SJ, Rossi MD, Lee SP. Disparities in functional recovery after dysvascular lower limb amputation are associated with employment status and self-efficacy. Disability and Rehabilitation. 2023 Jul 3;45(14):2280-7). These gaps in rationale should be expanded upon in a revised manuscript.

- We have added additional text to the discussion section to further reflect on why the explanatory power of the regression model was low.
- We have also clarified urban versus rural areas in the methods section (Note that living in either rural or urban settings did not significantly contribute to a change in mobility scores).
- As previously mentioned the concept of part-time versus full-time employment is not possible to capture in the Cambodian context where a significant proportion of the labor market is informal, unregistered and completly lacking social protections. We also argue that comparisons of employment status of this population against studies conducted in high income settings is contextually irrelevant.

VERSION 2 - REVIEW

**Reviewer**	**1**
**Name**	**Worsley, Peter**
**Affiliation**	**University of Southampton, School of Health Sciences**
**Date**	**27-May-2025**
**COI**	

All amendments have been made.
